# Intratumoral Hepatic Stellate Cells as a Poor Prognostic Marker and a New Treatment Target for Hepatocellular Carcinoma 

**DOI:** 10.1371/journal.pone.0080212

**Published:** 2013-11-20

**Authors:** Bin Sun, Xiaofeng Zhang, Xianshuo Cheng, Yu Zhang, Lei Chen, Lehua Shi, Zhenyu Liu, Haihua Qian, Mengchao Wu, Zhengfeng Yin

**Affiliations:** 1 Molecular Oncology Laboratory, Eastern Hepatobiliary Surgery Hospital, Second Military Medical University, Shanghai, China; 2 Department of Hepatic Surgery, Eastern Hepatobiliary Surgery Hospital, Second Military Medical University, Shanghai, China; The University of Hong Kong, China

## Abstract

Hepatic stellate cells (HSCs), a specialized stromal cytotype in the liver, have been demonstrated to actively contribute to hepatocellular carcinoma (HCC) development. However, the previous studies were performed using HSC cell lines, and the prognostic value of intratumoral HSCs (tHSCs) was unclear. Here we isolated tHSCs from fresh human HCC tissues, and analyzed the abilities of tHSCs to promote HCC progression by using in vitro assays for cell viability, migration and invasion as well as epithelial-mesenchymal transition (EMT) phenotype. 252 HCC patients who underwent hepatectomy were enrolled for analysis of tHSCs and E-cadherin expression in tumor tissues, and 55 HCC patients for analysis of tHSCs in tumor tissues and circulating tumor cells (CTCs) in blood. Prognostic factors were then identified. The results showed that coculture of tHSCs with HCC cells had a stronger effect on HCC cell viability, migration and invasion, accompanied with the acquisition of epithelial-mesenchymal transition (EMT) phenotype. In vivo cotransplantation of HCC cells with tHSCs into nude mice more efficiently promoted tumor formation and growth. Icaritin, a known apoptosis inducer of HSCs, was demonstrated to effectively inhibit tHSC proliferation in vitro and tHSC-induced HCC-promoting effects in vivo. Clinical evidence indicated that tHSCs were rich in 45% of the HCC specimens, tHSC-rich subtypes were negatively correlated either with E-cadherin expression in tumor tissues (r = -0.256, p < 0.001) or with preoperative CTCs in blood (r = -0.287, p = 0.033), and were significantly correlated with tumor size (p = 0.027), TNM staging (p = 0.018), and vascular invasion (p = 0.008). Overall and recurrence-free survival rates of tHSC-rich patients were significantly worse than those for tHSC-poor patients. Multivariate analysis revealed tHSC-rich as an independent factor for overall and recurrence-free survival. In conclusion, tHSCs provide a promising prognostic biomarker and a new treatment target for HCC.

## Introduction

Tumor microenvironment is also referred to as stroma, and basically consists of the extracellular matrix (ECM) and stromal cells [1]. The liver in particular consists of numerous specialized stromal cell types such as hepatic stellate cells (HSCs) and Kuffer cells. HSCs comprise up to 30% of the non-parenchymal cells in the liver [2], and represent a highly versatile cytotype [3]. It is well known that the majority of hepatocellular carcinoma (HCC) occur on a background of a chronic liver injury, and subsequent liver cirrhosis represents the main risk factor for developing HCC [4,5]. Following liver injury, quiescent HSCs (qHSCs) get activated and convert into highly proliferative myofibroblast-like cells, characterized by vitamin A lipid loss and α-smooth muscle actin (α-SMA) as well as desmin expressions [6]. Due to the vast remodeling of the extracellular matrix (ECM) and altered expression of growth factors, activated HSCs provide the cellular basis for the establishment of hepatic fibrosis and cirrhosis [7]. Upon HCC development, HSCs are markedly recruited into the stroma, activated under the control of tumor cells, and represent the prevalent cell type of the stromal cells [8-13]. Activated HSCs in turn act upon tumor cells, stimulating growth, migration, and invasion of hepatoma cells [14-19]. Coimplantation of HSCs and HCC cells into mice promoted tumor development [16,17]. However, all the cited studies were performed using either HSC cell lines or HSCs from normal livers.

Over the past decade, accumulating evidence has shown that the epithelial-mesenchymal transition (EMT), originally described during embryogenesis as a developmental process, is a pathological process contributing to cancer progression, particularly to invasion of the surrounding stroma, intravasation, and dissemination of circulating tumor cells (CTCs) into the peripheral blood [20]. While epithelial cells undergo EMT, loss of the epithelial marker E-cadherin and concomitant expression of distinct mesenchymal markers like vimentin play a vital role in this reversible transdifferentiation [20]

In the present study, we isolated intratumoral HSCs (tHSCs) from human HCC tissues, and found that coculture of tHSCs with HCC cells had a stronger effect on HCC cell behaviours, accompanied with the acquisition of EMT phenotype. Cotransplantation tHSCs into mice more efficiently promoted tumor formation and progression. Furthermore, icaritin, a confirmed apoptosis inducer of HSCs [21], was demonstrated to effectively inhibit tHSC proliferation in vitro and tHSC-induced HCC-promoting effects in vivo. Finally, clinical evidenc showed that tHSC-rich tumors were associated with the loss of E-cadherin expression, and involved in HCC cell invasion and CTC genaration. HCC patients with a tHSC-rich tumor were more likely to develop recurrence, and had a poor prognosis.

## Materials and Methods

### Ethics Statement

The use of human tissue samples and clinical data was approved by the Biomedical Ethics Committee of Eastern Hepatobiliary Surgery Hospital, Second Military Medical University (Shanghai, China). All patients provided the informed written consent. The animal welfare guidelines for the care and use of laboratory animals were followed and the experimental protocol was approved by the Animal Care Committee of Second Military Medical University. The mice were maintained in SPF environment of Experimental Animal Center and sacrificed by anesthetization.

### Patients and specimens

From May 2005 to June 2006, 252 patients with HCC who underwent curative partial hepatectomy were enrolled. All diagnoses were confirmed histopathologically. The patient demographics and clinicopathological factors are summarized in Table 1. All patients had postoperative follow-up by the same team of surgeons. A long-term follow-up was conducted as previously described [22]. The median follow-up was 43.0 months (range, 1.5–71.0 months).

**Table 1:** Correlations of tHSCs with clinicopathologic features in 252 HCC patients.

Variables	Total (n = 252)	tHSC-poor (n = 139)	tHSC-rich (n = 113)	P
Age (≤ 50/> 50 years)	111/141	66/73	45/68	0.223
Sex (male/female)	179/73	100/39	79/34	0.724
Hepatitis (HBV/HCV/none)	216/17/19	120/8/11	96/9/8	0.770
Serum AFP (≤ 400/> 400 ng/ml)	131/121	78/61	53/60	0.145
Liver cirrhosis (yes/no)	211/41	118/21	93/20	0.579
BCLC stage (0-A/B/C)	187/34/31	109/14/16	78/20/15	0.166
Tumor size (≤ 5.0/> 5.0 cm)	122/130	76/63	46/67	0.027
Tumor number (single/multiple)	190/62	110/29	80/33	0.126
TNM stage **^a^** (I/ II/ III)	81/103/68	52/46/41	29/57/27	0.018
Tumor encapsulation (yes/no)	118/134	72/67	46/67	0.079
Vascular invasion**^b^** (yes/no)	135/117	64/75	71/42	0.008

tHSCs: intratumoral hepatic stellate cells; HCC: hepatocellular carcinoma; AFP, alpha-fetoprotein; HBV, hepatitis B virus; HCV, hepatitis C virus; **^a^**the sixth edition of International Union Against Cancer (UICC) tumor-node-metastasis (TNM) staging system (2002); **^b^**Defined by findings on final pathololgical analysis.

### Primary human HSCs isolation, characterization and culture

Human HSCs were isolated, characterized and maintained in culture as previously described [23,24] except for fresh human tissues used. tHSCs were isolated from human HCC tissues, and qHSCs were isolated from distal normal liver tissue of hepatic hemangioma. To prepare culture-activated HSCs (aHSCs), qHSCs were cultured in uncoated plastic dishes in DMEM supplemented with 10% FBS for a total of 7 days [25]. For the production of HSC conditioned media (CM), HSCs were seeded into 6-well plates at a density of 5×10^5^ cells/well and cultured for 24 hours in DMEM supplemented with 10% FBS. After washed twice with serum-free DMEM, the cells were further incubated up to 24 hours in serum-free DMEM. The CM from tHSCs (tHSC-CM), qHSCs (qHSC-CM) and aHSCs (aHSC-CM) were separately collected. 

### Hepatoma cell culture and treatment

Human hepatoma cells PLC/PRF/5, Hep3B, and Huh-7 (American Type Tissue Culture Collection) were maintained as previously described [26]. For analysis of cell viability, adherent cells were cultured in the presence of tHSC-CM, qHSC-CM, and aHSC-CM, icaritin, or their combination. The icaritin solution (Yousi Biotech Inc., Shanghai, CHN) was prepared in dimethyl sulfoxide (DMSO) as previously described [21].

### Cell viability, migration and invasion assays

 Cell viability was assessed by using the MTT assay [21]. Confluent cell monolayers in 6-well plates were wounded by manual scraping with a sterile pipette tip [26]. The imagines were analyzed by Image J (NIH). Cell invasion assay was performed on Matrigel-coated transwell chambers (Corning, NY, USA) as previously described [26] except for the presence of different dilutions of HSC-CM. 

### Western blot analysis

Cellular proteins were electrophoresed and visualized as previously described [26] The imagines were analyzed by Quantity One (Bio-Rad, California, USA).

### Immunochemical staining 

Cells were grown to confluency on coverslips in 6-well dishes. For immunofluorescence, cells were consecutively incubated with unconjugated primary antibody and appropriate Cy3-labeled secondary antibodies (Beyotime, Nantong, CHN), and costained with DAPI (Sigma, MO, USA). For non-fluorescent immunocytochemical staining, the ABC method and DAB substrate (Maixin Bio, Fuzhou, CHN) were used as described below. For immunohistochemistry, the tissue sections with 4 µm thickness were consecutively incubated with unconjugated primary antibody and appropriate biotin-conjugated secondary antibodies (Maixin Bio), and the immunoreactivity was detected using the ABC kit and DAB substrate [27]. Immunoreactive score (IRS) proposed by Remmele and Stegner [28] was applied to immunoreactive evaluation. The assessment of the staining was conducted by two investigators independently.

### tHSC detection

tHSCs were identified by their intratumoral location, spindle-shaped morphology, and cytoplasmic expression of α-SMA. Glisson capsules, fibrous septa, collapsed parenchyma, and areas of vessels were not included [29]. According to the classification system described by Kellermann et al [30], the tHSC density was classified into 4 grades. For statistical analysis, grades (0) (1,2), were grouped together difined as tHSC-poor, grade (3) as tHSC-rich.

### HCC CTC detection

HCC CTC number was determined by a previously developed method named asialoglycoprotein receptor-based magnetic separation [31]. 

### TUNEL assay

TUNEL assay for apoptasis detction was performed according to the protocol of the one step TUNEL kit (Beyotime, Nantong, CHN) [32].

### Tumor xenograft experiments

Tumors were generated in BALB/c nude mice (Shanghai Laboratory Animal Center, Chinese Academy of Sciences, Shanghai, CHN) and calculated as previously described [33]. For icaritin treatmemt, icaritin dissolved in Olive oil was administered by gastric gavage, with a total volume of 1 ml/kg body weight per day, three times a week. 

### Statistical analysis

Data were expressed as median ± standard error or percent, and comparison between groups was made using the Student’s test and one-way ANOVA. Survival analysis was estimated by the Kaplan–Meier survival method. Variables with a p value of < 0.2 in the univariate analyses were further tested using multivariate Cox proportional hazards model. SPSS software (version 12.0 for Windows; SPSS Inc., Chicago, IL, USA) was used for all statistical analyses. A p value < 0.05 was considered statistically significant.

## Results

### Characteristics of isolated tHSCs from human HCC tissues

tHSCs could be isolated in substantial numbers with good viability (92-96%) and purity (95-98%). When plated on plastic, tHSCs spread with a spindle-shaped morphology, qHSCs presented a typical polygonal sharp, gradually showing muscle fibroblast-like change with prolonged cultured time (Figure S1A). Unlike qHSCs, almost all the isolated tHSCs were intensely positive for α-SMA and desmin (Abcam, Cambridge, UK) (Figure S1C) and loose vitamin A droplets without vitamin A autofluorescence (Figure S1B). 

### tHSC-CM more efficiently enhances proliferation, migration, and invasion of HCC cells in vitro

**Figure 1:**
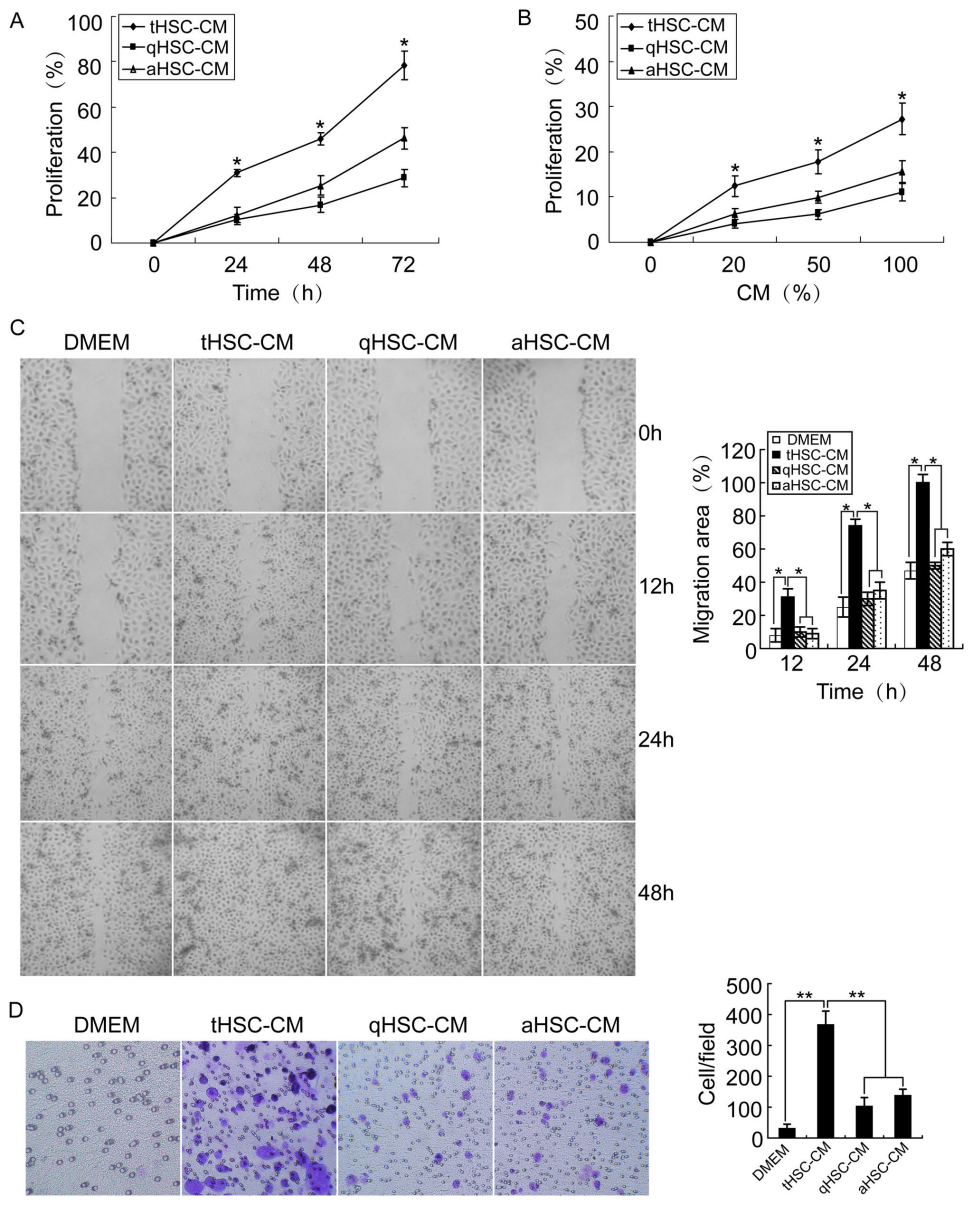
tHSC-CM promoted proliferation, migration and invasion of PLC/PRF/5 cells. (A and B) tHSC-CM significantly promoted HCC cell growth with time- and concentration-dependent manner. (C) Representative images of wound migration assays (left panel). Results are expressed as the percentage of the wounded area (right panel). (D) Representative images of invasion assays performed by transwell chamber (left panel). Results are expressed as the number of cells per field (right panel). *p < 0.05; **p < 0.01.

There was an increase in PLC/PRF/5 cell growth after a 24-hour incubation at different dilutions of tHSC-CM (*p < 0.05), and tHSC-CM gave a greater proliferative capacity than did qHSC-CM and aHSC-CM in a dose- and time-dependent manner (Figure 1A and 1B, *p < 0.05). tHSC-CM caused a significant increase in PLC/PRF/5 cell migration than qHSC-CM and aHSC-CM in wound healing assays (Figure 1C, *p < 0.05). Experiments using transwell chambers also indicated that tHSC-CM exhibited an increase in invasive cell number as compared to qHSC-CM and aHSC-CM at the same concentration (Figure 1D, **p < 0.01). The results were consistent with Hep3B and Huh-7 HCC cell lines in the different HSC-CM (data not shown).

### tHSC-CM induces EMT-like phenotype in HCC cells

**Figure 2:**
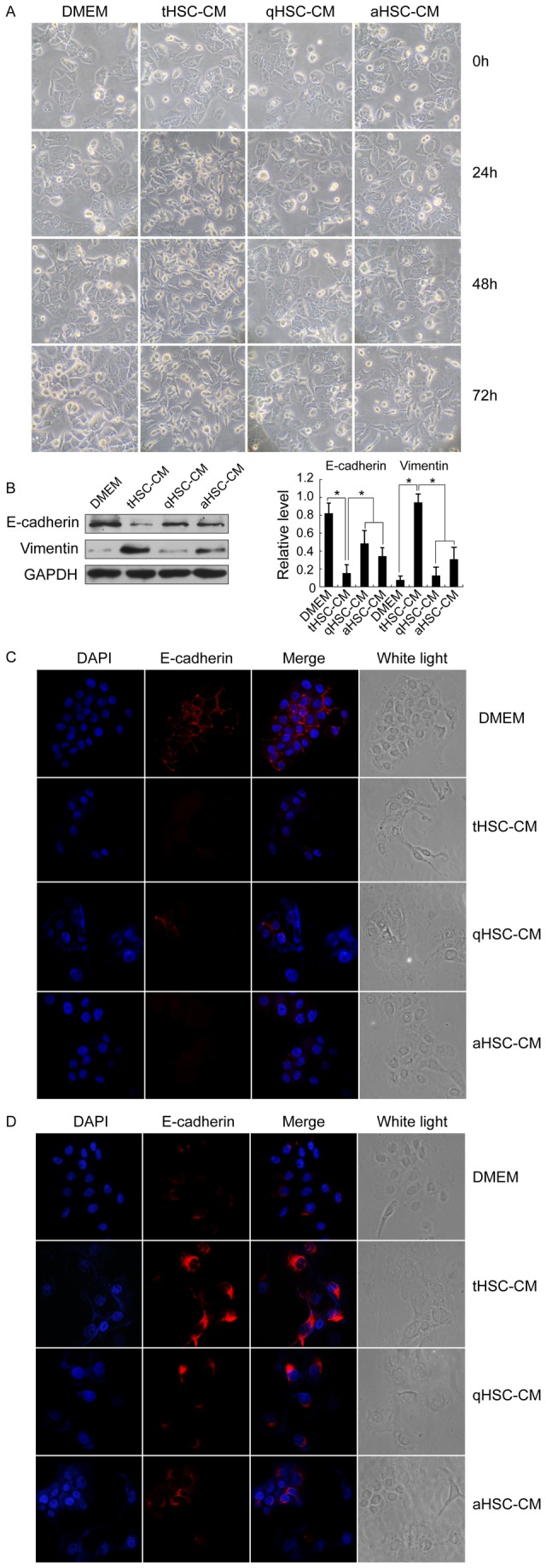
tHSC-CM induces EMT-like phenotype in HCC cells. (A) The morphology changes in PLC/PRF/5 cells (magnification, ×200). (B) Western blot analysis of E-cadherin and vimentin expressions in PLC/PRF/5 cells (left panel). Results are expressed as the fold value of protein levels compared with GAPDH (right panel). *p < 0.05. (C and D) Representative images of E-cadherin and vimentin expressions in PLC/PRF/5 cells by immunoflurescence staining (magnification, ×400).

Most cells on tHSC-CM stimulation for 48 hours underwent a dramatic change to a spindle-shaped, elongated morphology (Figure 2A), accompanied by decreased expression of E-cadherin (Abcam) and increased expression of vimentin (Abcam), either by immunoflurescence staining or by Western blot analysis (Figure 2B, 2C and 2D). Compared to qHSC-CM and aHSC-CM, tHSC-CM resulted in a much decreased expression of E-cadherin (Figure 2B, 2C and 2D). Parallel experiments with Hep3B and Huh-7 cells revealed changes similar to those in PLC/PRF/5 (Figure S2).

### Cotransplantation with tHSCs promotes HCC formation and growth in vivo

**Figure 3:**
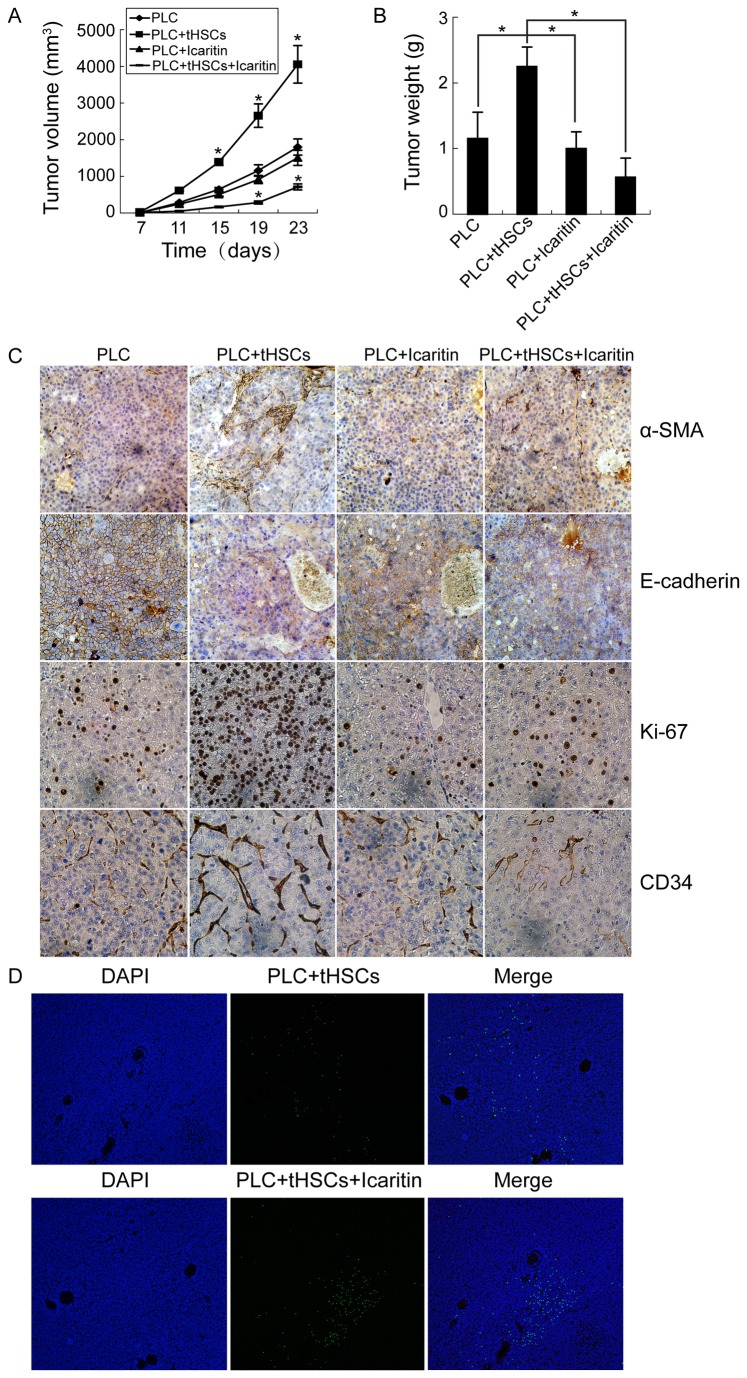
tHSCs promote HCC growth, and icaritin effectively inhibits tHSC-induced HCC-promoting effects in vivo. (A) The tumor volumes were monitored every 4 days up to 23 days after cell implantation. (B) The tumor weights at the end of experiments (*p < 0.05). (C) Representative immunohistochemical staining of α-SMA, E-cadherin, Ki-67, and CD34 expressions in different xenografts. (D) Representative images of TUNEL assay in different xenografts (magnification, ×200). PLC: PLC/PRF/5.

Within 7 days, visible xenografts were formed in 3 mice (5 mice/group) implanted with HCC cells plus tHSCs (5×10^5^ cells/mouse, respectively), and in 2 mice (5 mice/group) implanted with HCC cells plus aHSCs, but no xenograft tumor was generated in all the mice implanted with HCC cells alone (p < 0.05). When both the cells were increased to 5×10^6^ cells/mouse, all the mice implanted with either HCC cells alone or in combination with tHSCs developed tumors at 7 days, and the tumor volume between two groups didn’t displayed significantly difference (p > 0.05). However, on day 15 postinjection, the tumor volume from HCC cells plus tHSCs was much greater than that from HCC cells alone (Figure 3A, P < 0.05). Accordingly, the tumor weights from HCC cells plus tHSCs at time of killing were about 195% of the controls (Figure 3A and 3B). In xenografts, co-implantation of tHSCs led to increased tHSCs and decreased E-cadherin expression, accompanied with increased Ki-67 expression (Abcam) and higher microvessel density (CD34 antibody, Abcam) (Figure 3C). 

### Icaritin effectively inhibits tHSC proliferation in vitro and tHSC-induced HCC-promoting effects in vivo

Similar to the result from parallel experiments with activated HSC cell lines LX2 and HSC-T6 (rat) [21], icaritin also inhibited the proliferation of tHSCs with a dose-dependent manner within a concentration range of 5 μM to 20 μM, and the mean IC50 value was 18.09 μM at 48 hours. But, surprisingly, a concentration as high as 68.60 μM had limited effect on viability of HCC cell lines (Figure S4). 

According to the good growth inhibition activity and minimal toxicity [21], the 1 mg/kg daily dose of icaritin was subsequently used for the in vivo experiments. Treatment started on day 7 after cell implantation, when the HCC xenografts reached approximately 100 mm^3^. As shown in Figure 3A, on day 8 postreatment, the tumor volume from HCC cells plus tHSCs was much smaller compared to controls without treatment (p < 0.05), while that from HCC cells alone showed little change (p > 0.05). Accordingly, the tumor weight from HCC cells plus tHSCs at the end of treatment was approximately 25% of the controls without treatment, whereas that from HCC cells alone didn’t significantly reduced (p > 0.05). Icaritin treatment led to decreased tHSC density, blood microvessel density and Ki-67 expression, and increased E-cadherin expression and apoptotic rate in xenografts (Figure 3C, 3D). 

### tHSCs are associated with changes of E-cadherin expression in human HCC specimens

**Figure 4:**
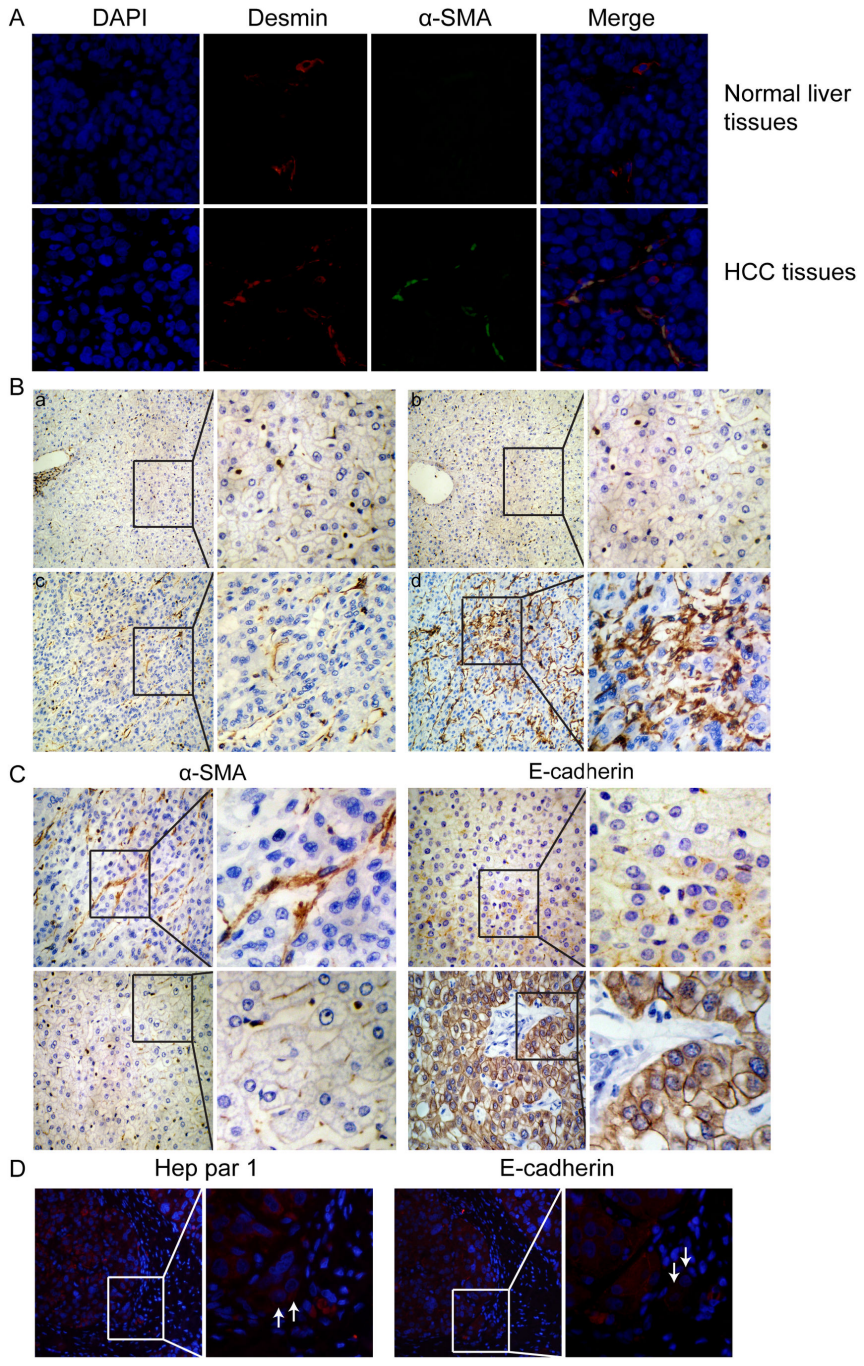
tHSCs are associated with E-cadherin expression, HCC cell invasion in human HCC specimens, and poor survival outcome. (A) Representative immunostaining of HSCs in normal liver tissues and HCC tissues with α-SMA and desmin antibodies. (B) Representative samples of the tHSC density determined by α-SMA immunostaining (magnification, ×200). Typical grades 0, 1, 2, and 3 are shown in a, b, c, and d, respectively. (C) Representative pictures of α-SMA and E-cadherin expressions in human HCC tissues detected by immunostaining (magnification, ×400). tHSC-rich is accompanied by deccreased E-cadherin expression in case 1 (upper panel), and tHSC-poor is accompanied by increase E-cadherin expression in case 2 (lower panel). (D) Immunofluorescence staining separately with Hep Par 1 and E-cadherin antibody on serial sections of one HCC sample (magnification, ×400). The white arrows indicated the tumor cells with both Hep Par 1-positive and E-cadherin-negative staining that have infiltrated from their nests into the surrounding stroma. (E) The impact of tHSCs on the survival of HCC patients. (a) tHSC-associated overall survival rate. (b) tHSC-associated recurrence-free survival rate.

qHSCs with desmin positive and α-SMA negative were mostly found in normal liver tissues, and aHSCs with both positive mostly in HCC tissues (Figure 4A). Examples with α-SMA and E-cadherin immunostaining in a human HCC tissue sample are shown in Figure 4B, 4C. A total of 252 clinical specimens were examined. tHSCs were rich in 45% (113/252) of the HCC specimens, and poor in 55% (139/252). The positive expression rate of E-cadherin expression was 46% (116/252) in HCC tissues, and 75% (189/252) in the surrounding tissues (p < 0.01). Their correlations are detailed in Table S1, and tHSCs were negatively correlated with E-cadherin expression (r = -0.256, p < 0.001). 

### tHSC-induced EMT is involved in HCC cell invasion and dissemination

In the above experiment, we especially observed that a few tumor cells in the tHSC-rich HCC tissues appeared to invade from cancer cell nests into adjacent tumor stroma. Hep Par 1 is a hepatocyte-specific antibody, usually used to recognize only liver-derived cells [34]. Further immunofluorescence staining on serial sections of HCC tissues showed that tumor cells infiltrated into the surrounding stroma were both Hep Par 1-positive and E-cadherin-negative staining (Figure 4D), indicating these cells being HCC cells and undergoing EMT. The correlation of tHSCs with CTCs was then analyzed in 55 HCC patients. Among them 24 were tHSC-rich in the HCC specimens, and 43 were positive for CTCs in the blood. The positive rate of CTCs was 92% (22/24) in tHSC-rich subtypes, and 32% (10/31) in tHSC-poor subtypes (p < 0.01). tHSC-rich subtypes were significantly associated with preoperative CTCs (r = -0.287, p = 0.033). 

### Correlations of tHSCs in HCC with clinicopathological factors

The clinicopathological variables between different HCC subtypes with tHSC-poor or tHSC-rich were compared in Table 1. The tHSC-rich subtypes were significantly correlated with tumor size (p = 0.027), TNM staging (p = 0.018), and vascular invasion (p = 0.008). 

### HCC subtypes with tHSC-rich have a poor prognosis

As shown in Figure 4E, HCC with tHSC-rich was associated with a significantly lower overall survival rate (log rank = 10.481, p = 0.001, Figure 4Ea) and recurrence-free survival (log rank = 5.453, p = 0.020, Figure 4Eb) than was HCC with tHSC-poor. Based on the univariate analysis, seven variables were selected to be further tested for multivariate analysis (Table 2). The results indicated that tHSC-rich was one of the independent favorable factors for overall and recurrence-free survival, as were BCLC stage, advanced TNM stage, and vascular invasion (Table 2).

**Table 2:** Univriate and multivariate analyses of the factors associated with survival and recurrence in 252 HCC patients.

	OS			RFS		
	Univeriate	Multivariate		Univeriate	Multivariate	
Factors	P	HR (95% CI)	P	P	HR (95% CI)	P
Age (≤ 50 vs. > 50 years)	0.337		NA	0.246		NA
Sex (male vs. female)	0.706		NA	0.912		NA
Hepatitis history (yes vs. no)	0.511		NA	0.453		NA
Serum AFP (≤ 400 vs. > 400 ng/ml)	0.042		NA	0.364		NA
Liver cirrhosis (yes vs. no)	0.458		NA	0.785		NA
BCLC stage (0-A vs. B-C)	< 0.001	3.439 (1.521-7.264)	< 0.001	< 0.001	2.531 (1.352-6.813)	< 0.001
Tumor size (≤ 5.0 vs. > 5.0 cm)	0.005		NS	0.027		NA
Tumor number (single vs. multiple)	0.033		NS	0.073		NS
TNM stage (I vs. II-III)	< 0.001	2.643 (1.262-6.735)	< 0.001	< 0.001	2.314 (1.107-6.074)	0.001
Tumor encapsulation (yes vs. no)	0.007		NA	0.029		NA
Vascular invasion (yes vs. no)	< 0.001	2.017 (1.013-5.247)	0.004	< 0.001	2.235 (1.022-4.316)	0.007
tHSCs (poor vs. rich)	< 0.001	1.885 (1.182-3.421)	0.013	0.020	2.033 (1.325-5.649)	0.042

Univeriate analysis: Kaplan-Meier method; multivariate analysis: Cox proportional hazards regression model.

Abbreviations: HCC: hepatocellular carcinoma; OS: overall survival; 95%CI: 95% confidence interval; RFS: recurrence-free survival; HR: hazard ratio; AFP: alpha-fetoprotein; NA: not adopted; NS: not significant.

## Discussion

Several previous experiments using HSC cell lines or HSCs isolated from normal livers have shown that aHSCs can actively contribute to the progression of liver carcinogenesis [35]. In the present study, we isolated tHSCs from HCC tissues, and demonstrated most of them in an activated state, providing evidence that HCC cells are able to activate tHSCs to create a supportive microenvironment. Compared with qHSC-CM and aHSC-CM, coculture of tHSCs had a stronger effect on HCC cell behaviours (Figure 1). As expected, tHSCs could also induce an EMT-like phenotype in HCC cells (Figure 2) and angiogenesis (Figure S3). Consistent with the in vitro results, cotransplantation of tHSCs into mice more efficiently promoted tumor formation and growth. To our knowledge, this is the first time to examine the involvement of primary tHSCs in tumor progression.

Stromal cells in the tumor are probably not completely identical to those in the normal tissues [36]. A recent study indicated that HCC-activated HSCs and aHSCs display a significantly different gene expression pattern, suggesting a more important role of HCC-activated HSCs in HCC progression [37]. HSCs are an important source of a broad spectrum of soluble factors within the liver [38,39]. Among them stand out transforming growth factor-α and -β (TGF-α, TGF-β), hepatocyte growth factor (HGF), Platelet derived growth factor (PDGF), and vascular endothelial growth factor (VEGF) [38,39]. These factors have proven to be essential for fostering an environment conducive to tumor cell growth, migration, and invasion, and also among the most potent EMT inducer of tumor cells and angiogenesis stimulator [40]. To explain why tHSCs had a stronger effect on HCC progression, we quantitatively determined some of these factors in tHSC-CM, qHSC-CM and aHSC-CM, and found that tHSCs produced higher levels of TGF-β, HGF, PDGF and VEGF than did qHSC and aHSC cells (Figure S5), further suggesting tHSCs as key regulators of the paracrine signaling between stromal and HCC cells. 

Similarly to what was observed with HCC cells in vitro, tHSC-rich pattern was strongly and inversely associated with E-cadherin expression in HCC tissues. Recent data suggest that during EMT, tumor cells seem to acquire more aggressive traits, and have an increased ability to invade into the surrounding tissue and migrate into the bloodstream [41]. In fact, we not only observed a few tumor cells in the tHSC-rich HCC tissues into adjacent tumor stroma (Figure 4C), but also found a significant association of tHSC-rich subtypes with preoperative CTCs, which are now considered to be an active source of HCC metastasis or recurrence [42]. These results suggest that tHSCs could promote HCC cell invasion and dissemination to the peripheral blood through EMT induction. 

A study conducted by Ju et al [43]. evaluated the prognostic potential of peritumoral activated HSCs in HCC patients, and found that peritumoral activated HSCs also contributed to high recurrence or death rates. However, little data have been published before on the association between tHSC density and HCC patient prognosis. Our results revealed that tHSC-rich was significantly associated with tumor size, TNM staging, and vascular invasion, indicating a critical role of tHSC-rich in the promotion of poor prognosis. Indeed, HCC subtypes with a tHSC-rich tumor had significantly shorter overall survival and recurrence-free survival than did HCC subtypes with a tHSC-poor tumor. Furthermore, multivariate analysis identified four prognostic factors influencing survival, among them tHSC-rich was an independent favorable factor for overall and recurrence-free survival. These results imply that HCC patients with a tHSC-rich tumor should receive close follow-up and novel interventional measures.

Because HCC is not very sensitive to chemotherapy, efforts to develop new drugs are shifting from systemic chemotherapy to therapeutic targeting of tumor-stromal interaction [44]. Our present study not only lighted the need for HSC-specific therapeutic strategies for HCC, but also provides icaritin as a potential new drug without overt toxicity for targeting tumor stroma. We have previously demonstrated that icaritin, an active monomer refined from the Chinese herb Herba Epimedii Sagittatum, can induce apoptosis in activated HSCs, and thereby ameliorate the development of liver fibrosis in rats [21]. In the current study, icaritin directly and specifically targets tHSCs, thereby abrogating all the cancer-promoting signaling pathways provided by the tHSCs. Obviously, this novel targeted therapy has its advantages over the strategies for targeting one molecular component produced by tHSCs, and appears to be promising and feasible.

In conclusion, our study demonstrates a stronger effect of tHSCs on HCC aggressiveness in vitro and in vivo, describes an important role for tHSCs in the promotion of HCC cell EMT and dissemination, and establishes a close correlation between tHSC-rich HCC subtypes and a poor prognosis. Effective inhibition of tHSC proliferation in vitro and tHSC-induced HCC-promoting effects in vivo by icaritin was also demonstrated. These results elaborate on tHSCs as key regulators of the paracrine signaling between stromal and HCC cells, and provide essential information for a promising prognostic biomarker and a new treatment target for future HCC management.

## Supporting Information

Figure S1 **Characterization of primary tHSCs.** (A) Images were taken on day 0, day 3, day 7, day 10 and day 14 of culture. (B) Vitamin A autofluoresence (upper panel) and light micrographs (lower panel) of primary tHSCs and qHSCs. (C) Cells were costained with a-SMA, desmin antibodies and DAPI.
(TIF)

Figure S2 **tHSC-CM induces EMT-like phenotype in Hep3B and Huh-7 cells.** The morphology changes were observed in Hep3B (A) and Huh-7 (B) cells under invert microscope (magnification, ×200). Representative images of E-cadherin and vimentin expressions in Hep3B (C) and Huh-7 (D) cells on HSC-CM stimulation by immunoflurescence staining. Nuclei were counterstained with DAPI (magnification, ×400). Western blot analysis of E-cadherin and vimentin expressions in Hep3B (E) and Huh-7 (F) cells that were treated with HSC-CMs (left panel). Results are expressed as the fold value of protein levels compared with GAPDH (right panel). Data are expressed as the means ± SD (*p < 0.05).
(TIF)

Figure S3 **tHSC-CM induces angiogenesis in vitro.** Human umbilical vein endothelial cells HUVECs (3×10^4^ cells per well) were seeded onto 48-well plates previously coated with matrigel matrix (100 μL/cm^2^) using serum-free DMEM. Endothelial tube formation was monitored after 6 hours in the presence of tHSC-CM, qHSC-CM and aHSC-CM (magnification, ×200). DMEM with 10% FBS and 3 ng/ml basic fibroblast growth factor were used as a positive control, and nonsupplemented DMEM was used as a negative control. Triplicate experiments were carried out. a: negative control; b: positive control; c: tHSC-CM; d: qHSC-CM; e: aHSC-CM.
(TIF)

Figure S4 **Impacts of icaritin on the viabilities of PLC/PRF/5, tHSCs, qHSCs, aHSCs, HSC-T6 and LX2 cells by MTT assay.** n.s: not significant; *p < 0.05; **p < 0.01 compared to other experimental groups.
(TIF)

Figure S5 **Cytokine concentrations (pg/ml) in different HSC-CMs by ELISA assays.** *p < 0.05 compared to other experimental groups.
(TIF)

Table S1 **Correlation of tHSCs with E-cadherin expression in 252 HCC tissues.**
(DOC)
