# Supplementary figures and images for: Intratumoral Hepatic Stellate Cells as a Poor Prognostic Marker and a New Treatment Target for Hepatocellular Carcinoma
Source: PLoS One. 2013 Nov 20;8(11):e80212. doi: 10.1371/journal.pone.0080212 (PMC3835887; doi:10.1371/journal.pone.0080212)

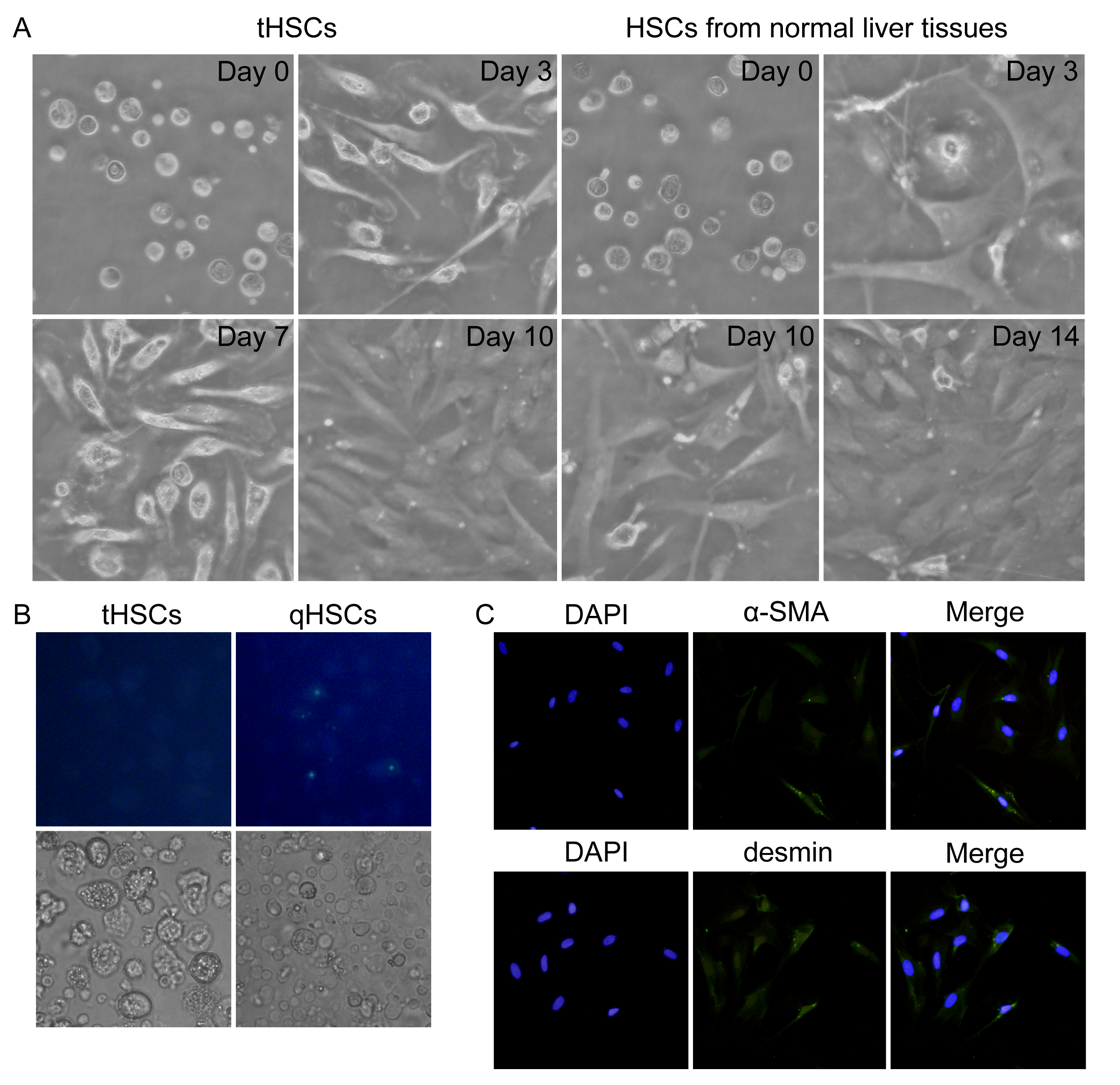

Supplement: Figure S1 — Characterization of primary tHSCs. (A) Images were taken on day 0, day 3, day 7, day 10 and day 14 of culture. (B) Vitamin A autofluoresence (upper panel) and light micrographs (lower panel) of primary tHSCs and qHSCs. (C) Cells were costained with a-SMA, desmin antibodies and DAPI. (TIF) [file pone.0080212.s001.tif]

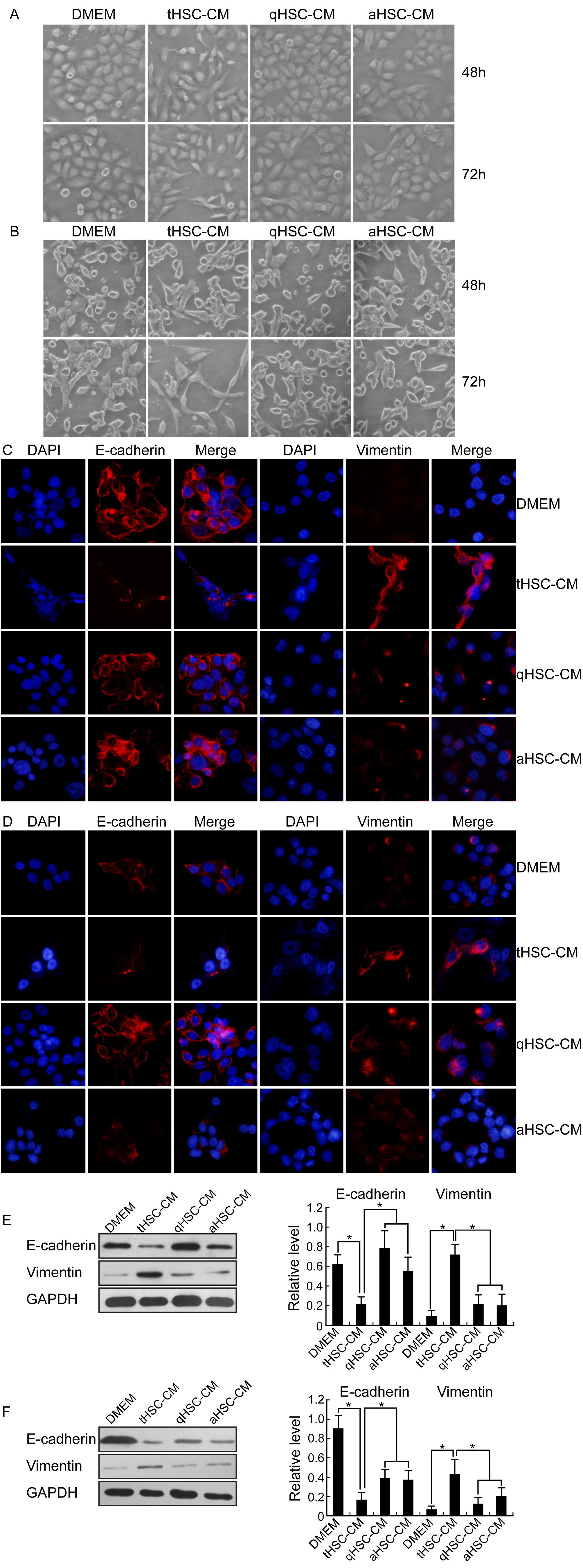

Supplement: Figure S2 — tHSC-CM induces EMT-like phenotype in Hep3B and Huh-7 cells. The morphology changes were observed in Hep3B (A) and Huh-7 (B) cells under invert microscope (magnification, ×200). Representative images of E-cadherin and vimentin expressions in Hep3B (C) and Huh-7 (D) cells on HSC-CM stimulation by immunoflurescence staining. Nuclei were counterstained with DAPI (magnification, ×400). Western blot analysis of E-cadherin and vimentin expressions in Hep3B (E) and Huh-7 (F) cells that were treated with HSC-CMs (left panel). Results are expressed as the fold value of protein levels compared with GAPDH (right panel). Data are expressed as the means ± SD (*p < 0.05). (TIF) [file pone.0080212.s002.tif]

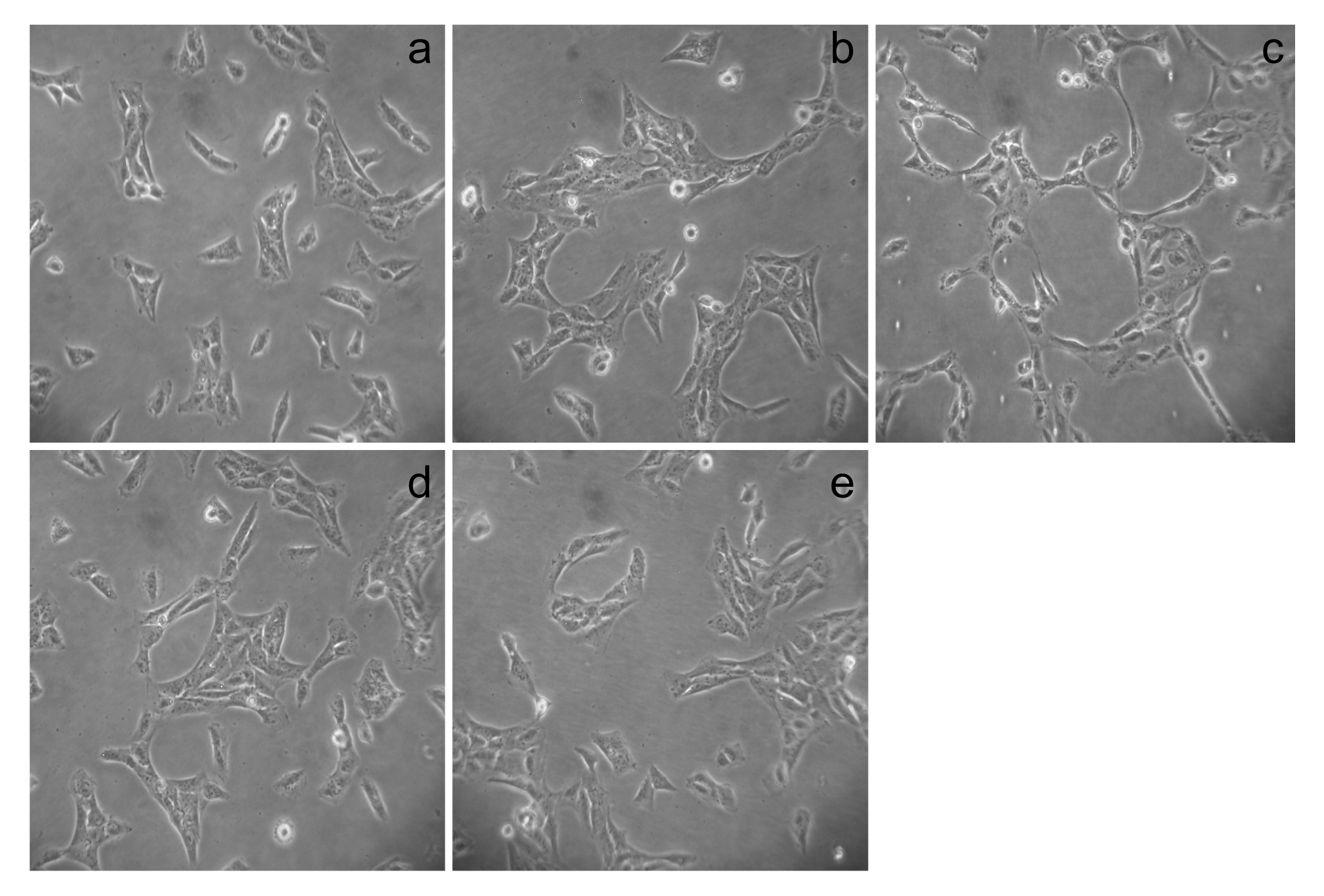

Supplement: Figure S3 — tHSC-CM induces angiogenesis in vitro. Human umbilical vein endothelial cells HUVECs (3×10^4^ cells per well) were seeded onto 48-well plates previously coated with matrigel matrix (100 μL/cm2) using serum-free DMEM. Endothelial tube formation was monitored after 6 hours in the presence of tHSC-CM, qHSC-CM and aHSC-CM (magnification, ×200). DMEM with 10% FBS and 3 ng/ml basic fibroblast growth factor were used as a positive control, and nonsupplemented DMEM was used as a negative control. Triplicate experiments were carried out. a: negative control; b: positive control; c: tHSC-CM; d: qHSC-CM; e: aHSC-CM. (TIF) [file pone.0080212.s003.tif]

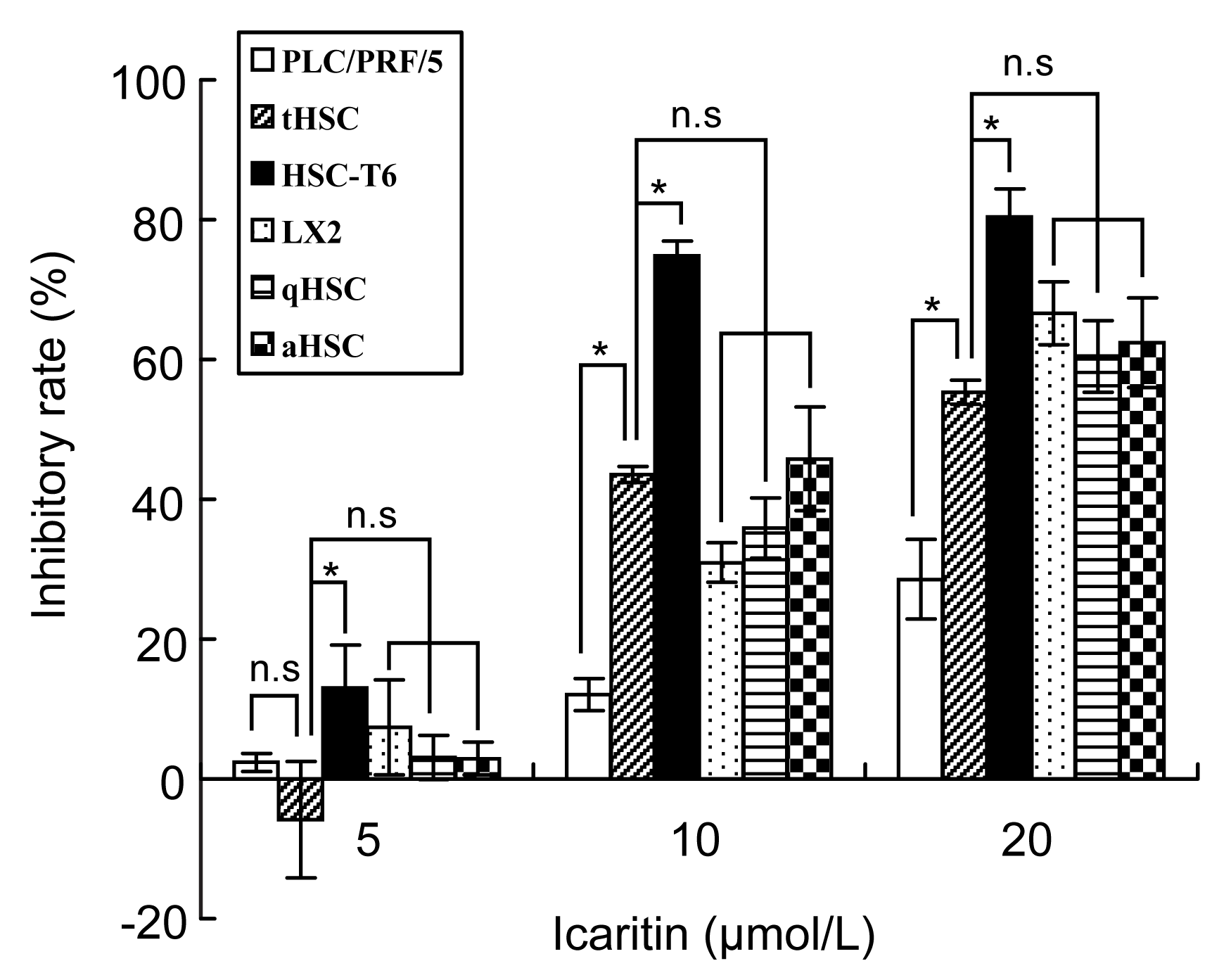

Supplement: Figure S4 — Impacts of icaritin on the viabilities of PLC/PRF/5, tHSCs, qHSCs, aHSCs, HSC-T6 and LX2 cells by MTT assay. n.s: not significant; *p < 0.05; **p < 0.01 compared to other experimental groups. (TIF) [file pone.0080212.s004.tif]

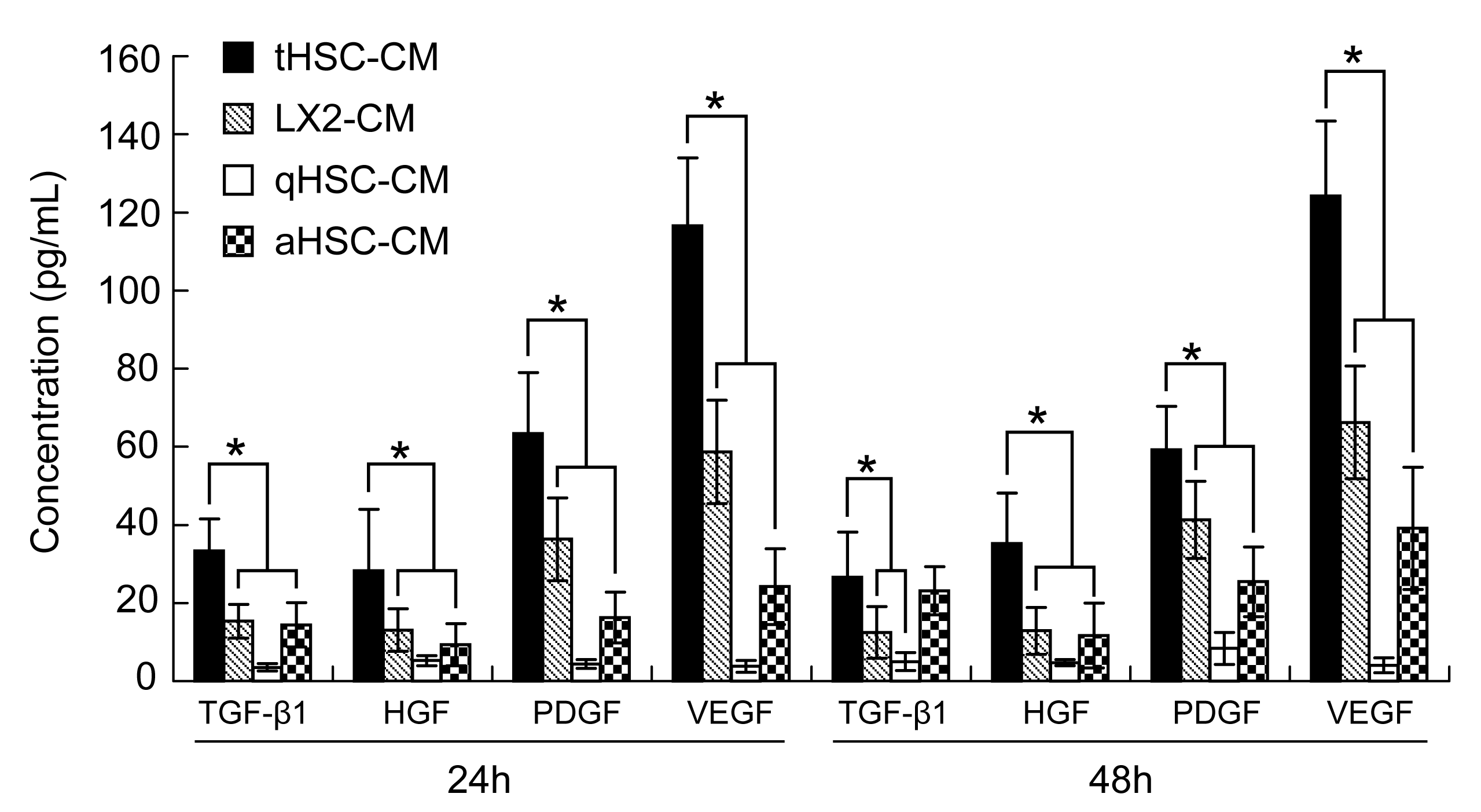

Supplement: Figure S5 — Cytokine concentrations (pg/ml) in different HSC-CMs by ELISA assays. *p < 0.05 compared to other experimental groups. (TIF) [file pone.0080212.s005.tif]
