# Supplementary material for: Intratumoral Hepatic Stellate Cells as a Poor Prognostic Marker and a New Treatment Target for Hepatocellular Carcinoma
Source: PLoS One. 2013 Nov 20;8(11):e80212. doi: 10.1371/journal.pone.0080212 (PMC3835887; doi:10.1371/journal.pone.0080212)
Supplement: Table S1 — Correlation of tHSCs with E-cadherin expression in 252 HCC tissues. (DOC) [file pone.0080212.s006.doc]

**Table S1 Correlation of tHSCs with E-cadherin**

**expression in 252 HCC tissues**

|  |  | tHSCs | |  |  |
| --- | --- | --- | --- | --- | --- |
|  |  | Poor | Rich | r | *P* |
| E-cadherin | Negative | 59 | 77 | -0.256 | < 0.001 |
|  | Positive | 80 | 36 |  |  |
